# A Framework for Addressing the Global Obesity Epidemic Locally: The Child Health Ecological Surveillance System (CHESS)

**Published:** 2008-06-15

**Authors:** Ronald C Plotnikoff, Penny Lightfoot, Carla Spinola, Gerry Predy, Linda Barrett

**Affiliations:** Centre for Health Promotion Studies, School of Public Health. Dr Plotnikoff is also affiliated with the faculty of Physical Education and Recreation, University of Alberta, and the Alberta Centre for Active Living, Edmonton, Alberta, Canada; Capital Health, Edmonton, Alberta, Canada; Capital Health, Edmonton, Alberta, Canada; Capital Health, Edmonton, Alberta, Canada; Centre for Health Promotion Studies, School of Public Health, University of Alberta, Edmonton, Alberta, Canada

## Abstract

Childhood obesity has reached epidemic levels in the developed world. Recent research and commentary suggest that an ecological approach is required to address childhood obesity, given the multidimensional nature of the problem. We propose a Canadian prototype, the Child Health Ecological Surveillance System, for a regional health authority to address the growing obesity epidemic. This prototype could potentially be used in other jurisdictions to address other child health issues. We present 8 guiding principles for the development and implementation of a regional framework for action.

## Introduction

Childhood obesity (defined here as overweight or obese according to Centers for Disease Control and Prevention guidelines) has reached epidemic levels (1-3). Globally, an estimated 22 million children under the age of 5 years are overweight (4), and 10% of those aged 5–17 are overweight or obese (5). During the last 2 decades, prevalence rates in the 5–17 age group have increased by 0.5% per year in the United States and Brazil, and by almost 1% per year in Canada, Australia, and the United Kingdom (5). In the United States, prevalence of obesity among children aged 6–11 years has more than doubled since the 1960s (4). In Canada in 1981, 11% of boys and 13% of girls were overweight or obese; within 15 years, these figures reached 33% and 27%, respectively (5). Given the seriousness of health risks associated with obesity, action is required immediately.

Obesity has a multifactor etiology, and its complexity merits broadening the traditional interventions to include the underinvestigated environmental aspects and the lack of a coordinated and interdisciplinary research agenda (6). Current prevention and treatment models for childhood obesity focus primarily on nutrition, physical activity, and body composition with little attention to environmental influences (7). Studies based on these models reveal limited impact on the overall problem (2,3). Novel approaches are required to address the magnitude of the childhood obesity health issue, approaches that avoid the temptation to implement one-time projects and individual-level research and that mobilize knowledge across ecological levels from research to practice (8).

## The Framework for Action on Healthy Body Weight in Children

A Canadian health authority, motivated by the continuing increase in obesity prevalence rates in Canada (9,10), sought to develop a regional framework for action by identifying the local scope of the problem and taking evidence-based action. Public health officials from Capital Health, a health region in Edmonton (Alberta, Canada), in collaboration with researchers at the University of Alberta, reviewed the literature and surveyed key national (n = 14) and international (n = 4) researchers and regional stakeholders (n = 37) to develop a framework for action on obesity in children. The survey was conducted by either a telephone or face-to-face interview. Joint leadership and teamwork among public health and academic representatives was identified from the outset as essential to the framework's success. Such collaboration and partnership create a rich environment to enhance research relevance and capacity without the need for an intermediary, fostering conditions for ready uptake of new knowledge. Given the multifactor etiology of childhood obesity (including genetic, physiological, behavioral, and environmental factors) (7), the multilevel ecological exploratory survey of regional, national, and international experts was used to gather a wide range of perspectives on the current knowledge base.

An ecological framework is a systems model that views behavior patterns — of individuals or aggregates — as the outcome of interest. Behavior is seen to be influenced by several factors (11-13):

- Personal factors of the individual: genetic, physiological/biomedical, cognitive, attitudinal, behavioral, and developmental history.
- Interpersonal processes and primary groups: formal and informal social network and support systems (i.e., family, peers, neighbors, friends).
- Institutional factors: social institutions with organizational characteristics, plus formal and informal rules of operation (i.e., norms, culture, structures, rules, regulations, incentives in schools and other institutions that relate to children).
- Community factors: relationships among institutions and organizations, and informal networks within defined boundaries (i.e., area economics, media, community services, neighborhood organizations, folk practices, municipal structures, formal and informal leadership).
- Public policy: municipal, provincial, and national laws and policies (i.e., legislation, policy, taxes).
- Physical environment: built and natural aspects of the environment (i.e., facilities, playgrounds, parks, trails; safety factors; and geographical aspects such as climate).

In this framework, interrelationships between the individual and his or her environment as well as interactions within and between the various ecological levels are considered. Individuals interact with the environment in multiple local settings, or microenvironments (e.g., homes, neighborhoods, schools, workplaces) (14). These microenvironments, in turn, are influenced by broader sectors, or macroenvironments (e.g., education and health systems, all levels of government, food industry) (14). Hence, this model provides a framework through which the interaction of the child's individual dimensions (i.e., biomedical, attitudinal, and behavioral) with the multiple components of his or her life context (i.e., social, organizational, community, public policy, and physical environments) can be examined.

The Framework for Action on Healthy Body Weight in Children (the Framework) (Figure) emerged from a synthesis of both the literature (15-23) and our survey findings. The Framework depicts the key ingredients required to advance knowledge and guide action by those with clinical and population health accountabilities in the prevention and treatment of obesity in children. The Framework is based on the four-step approach of the World Health Organization (24) for action on a public health problem with a multifactor and complex etiology: 1) surveillance (i.e., What’s the problem?), 2) risk factor or condition identification (i.e., What’s the cause?), 3) intervention and evaluation (i.e., What works?), and 4) implementation (i.e., How do you do it?). Other components — leadership, will to act, and infrastructure — are the cornerstones of capacity to implement these approaches (25,26).

**Figure.**
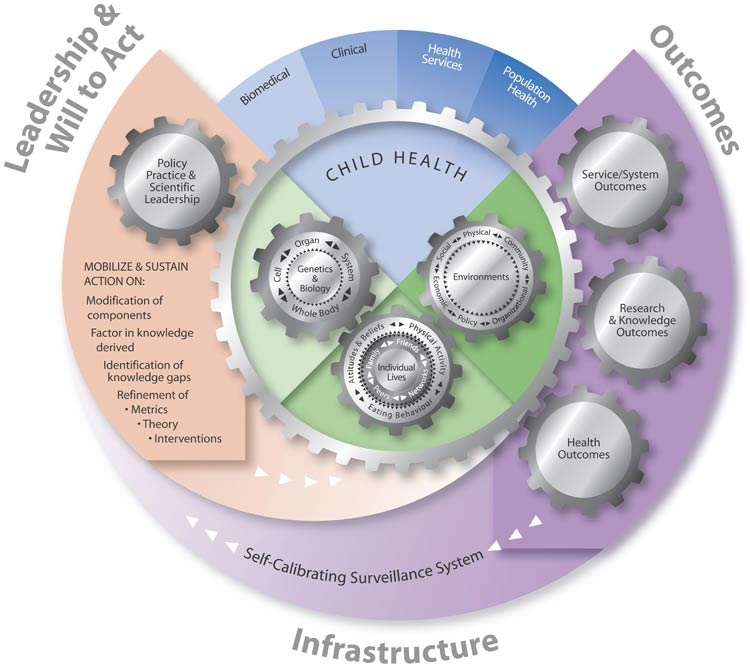
Framework for Action on Healthy Body Weight in Children

**Table:** 

### Child Health Ecological Surveillance System (CHESS): a Canadian prototype

The Framework depicts leadership and will to act (25,26) as igniting and driving a research program to enhance system capacity for addressing obesity in children. The engine of the Framework is the Child Health Ecological Surveillance System (CHESS). This is being developed as a regional prototype to provide ongoing valid and reliable health information about childhood obesity. The surveillance system incorporates a multidisciplinary ecological approach and represents the infrastructure needed for effective action. The prototype is portable to other jurisdictions and to other child health issues; it is a local approach to a global issue. The Framework reflects a strong integration of research and practice at the regional health authority level. This level of specificity is required to identify the extent of the local obesity problem. CHESS infrastructure allows surveillance for childhood obesity on a range of indices from individual to environmental influences, thereby capturing bidirectional feedback loops across and between ecological levels (11). The Figure and the bullet list of behavioral influences in the preceding section detail these variables, which align to the 4 pillars (i.e. biomedical, clinical, health services, and population health) of the Canadian Institutes for Health Research, Canada's major health and medical research funding agency.

The Framework further reflects service and system outcomes as well as research and knowledge outcomes (depicted on the right side of the Framework in the Figure). This information is then provided back to local decision makers so they can modify the surveillance system as needed; identify knowledge gaps; and refine metrics, theory, and interventions (see the left side of the Framework in the Figure). Ongoing collection of local data on core measures at multiple ecological levels will 1) provide ongoing regional prevalence data; 2) allow for the testing of theories related to secular trends in childhood obesity; and 3) guide the development and evaluation of treatment and prevention interventions by providing reliable information.

To summarize, action is initiated and ongoing in the Framework via joint leadership (practice and academic). This leadership mobilizes the will to act on gaps in knowledge, identified through CHESS, related to healthy body weight in children. Feedback loops via leadership and will to act generate the ongoing development and recalibration of CHESS metrics. Likewise, feedback loops help shape the dynamic evolution of the research agenda, which builds on the knowledge gained and mobilized along an overarching ecological framework of research and practice.

### Guiding principles for action

We identified 8 guiding principles for the CHESS process to guide health issues such as childhood obesity.

1. **Establish a diverse team of highly motivated and strategically placed individuals to guide CHESS through identification, intervention, surveillance, recalibration** *.* Interdisciplinary teamwork is key in addressing complex, multifaceted health issues affecting population health such as childhood obesity. Integration of scientific expertise with health practitioner and decision maker perspectives is essential from project conceptualization through to evaluation.
2. **Develop a local jurisdictional focus in which to apply the Framework.** A local jurisdictional focus will serve as a catalyst to concentrate multidisciplinary and multisector stakeholder activity. Making a strategic decision to collaborate with key decision makers and existing practitioners in the health region promotes prompt, focused, and sustained activity over the long term. Achieving a local jurisdictional focus also requires access to data on local prevalence rates of pediatric obesity. Local data will help determine whether the "epidemic" is manifesting locally, and ensures local accountability while demanding that decision making be evidence-based.
3. **Embrace differences to enhance the knowledge exchange quotient.** Naturally inherent tensions between the diverse goals and perspectives of practitioner, decision maker, and researcher need to be addressed. Differences can be perceived as strengths rather than problems. Valuing differences can help to avoid "group-think" and to catalyze synergistic action for the ultimate benefit of children.
4. **Recognize the importance of the three Ps — positioning, profile, and politics.** Those directing the surveillance process are encouraged to position it as an asset to those who have a mandate for child health (key stakeholders in government such as ministers and representatives of the health, education, and community sectors). Recognize that both the outcome and process depend on the public profile created and the local political agendas operating in the region. Addressing all three Ps will have a substantial impact on both the strength and sustainability of the initiative.
5. **Adopt an ecological perspective because complex issues, such as pediatric obesity, require complex responses.** Live with the complexity — don't try to minimize it. The diverse obesogenic environment (e.g., fast food availability, supersized portions, advertising, urban design, transportation policy) cannot be ignored; therefore, action must be multifaceted. A focus on the interrelationships between individuals and their environments allows for examination of the interaction of children with the components of their lives (i.e., social, organizational, community, public policy, and physical environments) with respect to obesity.
6. **Address issues of feasibility, sustainability, and accountability.** Build the surveillance system into existing population health initiatives operating in the region. Building on existing platforms allows supportive resources, such as human and financial resources, to flow more easily.
7. **Encourage accessibility to the information generated.** Broad access to the findings generated by CHESS is paramount for knowledge advancement. Partnership from the outset ensures knowledge dissemination and update and can ultimately save time.
8. **Think theoretically and act strategically.** Establish a system for responsive local and ongoing surveillance. The system should provide valid and reliable prevalence data related to childhood obesity and support the testing of theories related to secular trends in pediatric body weight. Although the focus is obesity, the ultimate goal of the CHESS process is healthy children.

CHESS represents a prototype for addressing childhood obesity through a local approach, with possible generic applications and global implications. The process and guiding principles are intended to be relevant in diverse regional settings, and where possible they should be used in conjunction with broader, coordinated provincial or state and national systems (e.g., using congruent core metrics). We suggest employing as many as possible of the dimensions of the framework and its guiding principles in developing such regional surveillance systems. However, such a framework may not be fully generalizable to or logistically possible for every regional context and may require modification. The process also will require tailoring to effectively capture the specific characteristics of the regional context (e.g., using local organizational, community, policy, and environmental metrics).

Our preliminary results from a feasibility study conducted with a convenience sample of 31 professionals in 3 Alberta cities revealed a strong need for a system like CHESS and support for it from pertinent stakeholder groups. We found that information on physical activity and nutrition is available at the organizational, community, macro-policy, and environmental levels. For example, municipalities through their parks, recreation, community services, and planning departments have general information regarding use of parks, trails programs, and facilities; they also have information on resource distribution and use at the neighborhood and city levels. However, data for the individual and social levels are much more limited and difficult to obtain and link to the system. Individual- and social-level metrics (e.g., body mass index [BMI], physical activity behavior, nutrition behavior) appear to be either 1) nonexistent or 2) unable to be shared or linked because of confidentiality issues with current databases, the use of incongruent measures, or the use of inconsistent data formats. Establishing compatible data formats that will easily and effectively link both cross-sectional and longitudinal measures within and between all ecological levels is imperative for the CHESS process, but is still rather underdeveloped within existing regional systems, especially in small municipalities. However, potentially successful and innovative strategies do exist; regional health authorities are developing protocols to track objective assessments of children's BMI along with parents' reports of their children's physical activity and nutrition behavior, as part of vaccination programs conducted in health care settings and schools. With its potential to formalize and speed such efforts, the multilevel CHESS process will, we hope, direct ongoing surveillance, theoretical and applied research, and public health initiatives aimed at ameliorating the childhood obesity epidemic.
